# A High-Temperature Stable Ohmic Contact Process on Lightly Doped n-Type 4H-SiC Based on a W/C Multilayer Structure

**DOI:** 10.3390/mi16121408

**Published:** 2025-12-15

**Authors:** Yu Zhou, Fengyu Du, Qingwen Song, Xiaoyan Tang, Hao Yuan, Chao Han, Chunfu Zhang, Yuming Zhang

**Affiliations:** 1 School of Microelectronics, Xidian University, Xi’an 710071, China; zhouyu01@xidian.edu.cn (Y.Z.); xytang@mail.xidian.edu.cn (X.T.); haoyuan@xidian.edu.cn (H.Y.); cfzhang@xidian.edu.cn (C.Z.); zhangym@xidian.edu.cn (Y.Z.); 2 Xidian-Wuhu Research Institute, Wuhu 241000, China; hanchao@xdwh-inst.com

**Keywords:** 4H-SiC, ohmic contacts, high temperature, thermal stability

## Abstract

In this paper, we propose a novel method for fabricating high-thermal-stability Ohmic contacts on 4H-SiC using a low-doping-concentration (2.5 × 10^15^ cm^−3^) n-type epitaxial layer. The method employs a tungsten/carbon (W/C) multi-nanolayer stacked structure combined with a 1200 °C rapid thermal process (RTP). The fabricated Ohmic contacts achieve a specific contact resistance *ρ*_c_ of 2.53 × 10^−4^ Ω·cm^2^ at room temperature (RT) and 1.29 × 10^−5^ Ω·cm^2^ at 500 °C. Furthermore, they exhibit excellent long-term operational reliability, maintaining stable performance during a 500 °C high-temperature test for 100 h in air without significant degradation. This method eliminates the need for ion implantation, avoiding lattice damage and reducing fabrication cost. The demonstrated thermal stability is highly desirable for elevated-temperature SiC-based devices and integrated circuits.

## 1. Introduction

As the backbone of the electronics industry, semiconductor devices and circuits inevitably operate in various high-temperature environments (>500 °C), such as vehicle exhaust sensing, interplanetary spacecraft, aerospace energy-management systems, and engine monitoring [1,2,3]. Traditional silicon-based electronics cannot function properly above 200 °C due to intrinsic material limitations [4]. Although silicon-on-insulator (SOI) technology can increase the operating temperature to approximately 300 °C [5], this is still far below the target of greater than 500 °C. Owing to its wide bandgap and extremely low intrinsic carrier concentration, silicon carbide (SiC) has become one of the most promising candidates for high-temperature applications [6]. As a critical process module in SiC integrated circuits (ICs), the thermal stability of Ohmic contacts is especially important.

Nickel-based (Ni/SiC) Ohmic contacts are technologically mature and exhibit low specific contact resistance (*ρ*_c_) [7]. However, the formation of these contacts relies on interfacial reactions between Ni and SiC, and the weak affinity of Ni for carbon (C) becomes a critical limitation during this process. During high-temperature operation, free carbon decomposed from SiC tends to accumulate at the metal/4H-SiC interface, forming Kirkendall voids, which cause severe thermal instability and degrade bonding reliability [8,9]. To address this problem, metals with strong carbon affinity, such as Ti and W, have been adopted as carbon-diffusion barrier layers [10,11]. Noble metals, including Pt, Au, and Ta, have also been used to enhance thermal stability [11]. Liu et al. [12] investigated TiW/Ni/SiC contacts on n-type 4H-SiC (5 × 10^18^ cm^−3^) and demonstrated improved thermal stability, maintaining Ohmic behavior after 100 h at 400 °C in N_2_. Lee et al. [13] reported Pt/Ti/TiW/SiC contacts on heavily doped 4H-SiC (1.1 × 10^19^ cm^−3^), which remained stable after 520 h at 500 °C in an oxidizing environment. Jang et al. [14] developed W/WC/TaC contacts on 6H-SiC (7.8–8.1 × 10^18^ cm^−3^), achieving outstanding stability after 1000 h at 600 °C in vacuum.

Previous studies have demonstrated that introducing additional barriers or noble-metal layers can improve high-temperature reliability; however, these works primarily focus on heavily doped SiC substrates. Very few studies investigate direct Ohmic contact formation on lightly doped SiC, despite its importance in a range of practical devices. In addition to photoconductive semiconductor switches [15], lightly doped 4H-SiC is widely employed as the drift layer in high-voltage unipolar power devices, including Schottky barrier diodes (SBDs), junction barrier Schottky (JBS) diodes, and SiC MOSFETs, where low-carrier concentration is essential to sustain high breakdown voltages. Achieving low-resistance Ohmic contacts in this doping regime is therefore critical to minimizing conduction loss and improving device efficiency. Moreover, forming contacts directly on lightly doped epitaxial layers reduces process complexity, minimizes the thermal budget, and avoids ion-implantation-induced damage, offering a more fabrication-friendly approach for next-generation SiC devices.

To further exploit the high-temperature potential of SiC, this work proposes a novel method for fabricating Ohmic contacts on a low-doping-concentration (2.5 × 10^15^ cm^−3^) n-type 4H-SiC epitaxial layer without ion implantation. The resulting contacts exhibit good specific contact resistance and excellent thermal stability up to 500 °C.

## 2. Experimental Procedure

**Figure 1:**
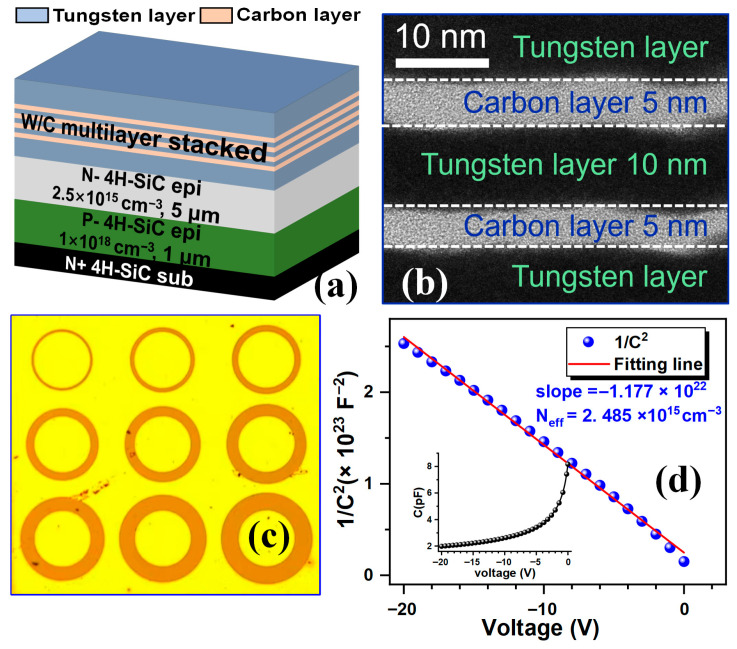
(**a**) Three-dimensional schematic and (**b**) cross-section HRTEM image of the W/C multi-nanolayers stacked structure. (**c**) Top view photograph of the c-TLM pattern. (**d**) 1/C^2^ as a function of the voltage.

Figure 1a shows the three-dimensional schematic of the W/C multi-nanolayer stacked structure, while Figure 1b presents the corresponding high-resolution transmission electron microscopy (HRTEM) image. The stacked structure consists of five pairs of W (~10 nm) and C (~5 nm) layers, forming a total of ten layers. The circular transmission line model (c-TLM) structure used to extract the contact resistance is shown in Figure 1c. The inner-circle radius (R) is 150 μm, and the gap space (d) is from 10 μm to 90 μm.

The Ohmic contacts were fabricated on a 5 µm thick n-type 4H-SiC epitaxial layer that was in situ doped with nitrogen to a concentration of 2.5 × 10^15^ cm^−3^ during the growth process and grown on a 300 µm thick n^+^-type 4H-SiC substrate. The effective doping concentration (Neff) extracted from the 1/C^2^-V curve [16] (Figure 1d) is about 2.485 × 10^15^ cm^−3^. A p-type 4H-SiC buffer layer was inserted between the n^+^ substrate and n-epitaxial layer to prevent current spreading into the substrate and improve resistance–extraction accuracy. The c-TLM patterns were fabricated by using standard lithography. After lithography, the W/C multi-nanolayers were deposited by sputtering and patterned using a lift-off process. The samples were then annealed in N_2_ using RTP for 600 s. For comparison, pure W contacts were fabricated under identical conditions.

## 3. Results and Discussion

**Figure 2:**
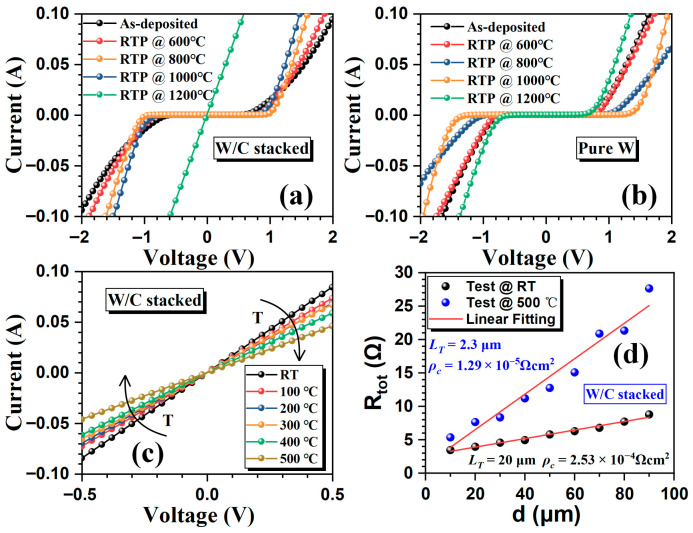
Typical *I–V* characteristics (at 20 μm gap) of the (**a**) W/C stacked contacts and (**b**) pure W contacts at different RTP temperature. (**c**) Typical *I–V* characteristics of the after 1200 °C RTP W/C stacked Ohmic contacts at different temperatures. (**d**) The total resistance of the W/C stacked Ohmic contacts as a function of the gap spacing at RT and 500 °C.

Figure 2a shows the *I–V* characteristics (at 20 μm gap) of the W/C stacked contacts annealed at different temperatures. Ohmic behavior is achieved only after RTP at 1200 °C. In contrast, the pure W contacts fail to form Ohmic behavior under the same conditions (Figure 2b). Figure 2c shows the *I–V* characteristics of the W/C stacked contacts Ohmic from RT to 500 °C. As the operating temperature increases, the *I–V* curve remains linear throughout, implying that the fabricated Ohmic contact can work at such high temperatures. To evaluate the quality of the fabricated W/C stacked Ohmic contacts, the *ρ*_c_ is extracted by the c-TLM [17]. Figure 2d presents the R_tot_ as a function of the gap space at RT and 500 °C. The *ρ*_c_ is 2.53 × 10^−4^ Ω·cm^2^ at RT and drops 1.29 × 10^−5^ Ω·cm^2^ at 500 °C.

**Figure 3:**
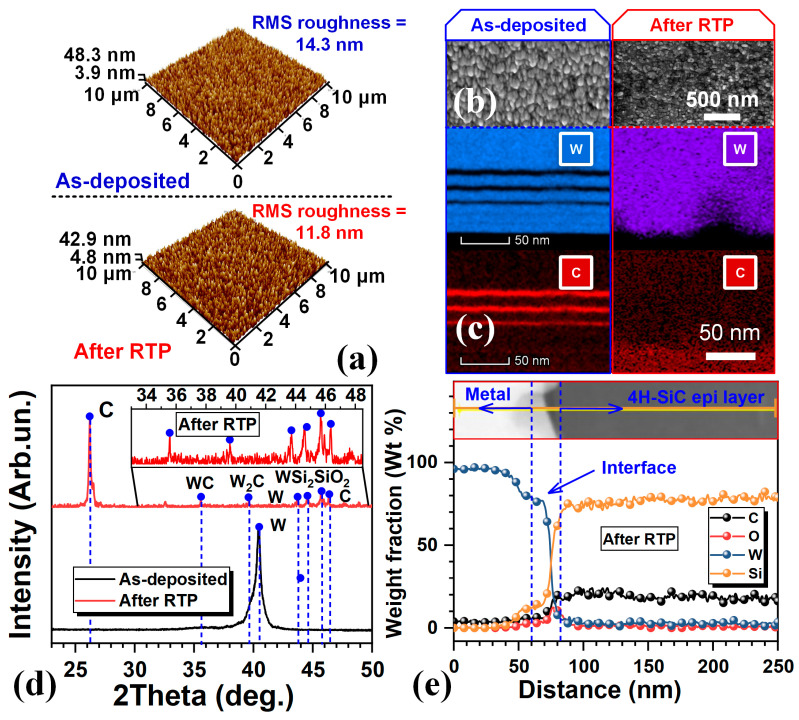
(**a**) Representative AFM surface 3D images, (**b**) top view SEM image, (**c**) EDX mapping of W and C elementals, and (**d**) GIXRD patterns of the as deposited sample and after RTP sample. (**e**) EDX depth profiles of the after RTP sample.

Surface morphology is critical for subsequent wire-bonding processes. The surface atomic force microscope (AFM) 3D images and top view SEM image of the as deposited and after RTP samples are shown in Figure 3a,b. The root mean square (RMS) roughness is 14.3 nm as deposited and 11.8 nm after RTP, and the crystallization is also more uniform and finer after RTP. This indicates that 1200 °C RTP does not affect the surface morphology of the contact metal.

The energy dispersive X-ray spectroscopy (EDX) mapping (Figure 3c) confirms metal delamination disappeared after RTP and seemingly reacted during annealing. The Grazing Incidence X-ray diffraction (GIXRD) patterns (Figure 3d) reveal multiple new phases after RTP. The as-deposited sample only detected one W (110) peak at 40.3°. In contrast, after RTP, seven diffraction peaks appear at 26.1°, 35.6°, 39.6°, 43.9°, 44.4°, 45.3°, and 46.3°, corresponding to C, WC, W_2_C, W, WSi_2_, SiO_2_, and C, respectively [18]. The emergence of these W–C and W–Si phases, particularly WC, W_2_C, and WSi_2_, is considered to play a crucial role in establishing Ohmic contact behavior, as these interfacial compounds enhance carrier transport pathways and reduce the effective barrier height at the metal/4H-SiC interface. The SiO_2_ peak originates from slight oxidation of exposed SiC during processing.

To investigate the new WC alloys distributed, Figure 3e shows the EDX depth profiles of the after-RTP sample. It can be observed that, although carbon is already present in the multilayer W/C layers, additional carbon from the SiC substrate also participates in the reaction. During annealing, W diffuses toward the SiC side, while carbon released from the SiC and multilayer W/C layers simultaneously diffuses toward the metal/4H-SiC interface, resulting in the formation of WC phases at the interface and inside the metal. J. Rogowskia and A. Kubiak [19] observed that W and C react when annealed at 1200 °C to WC spikes and toward SiC bulk substrate, which is the main factor for the formation of ohmic contacts. The presence of the spikes increases the electric field at the interface to enhance carrier transport [20,21]. In addition, the WSi_2_ at the interface may contribute to the Ohmic contact forming [22].

**Figure 4:**
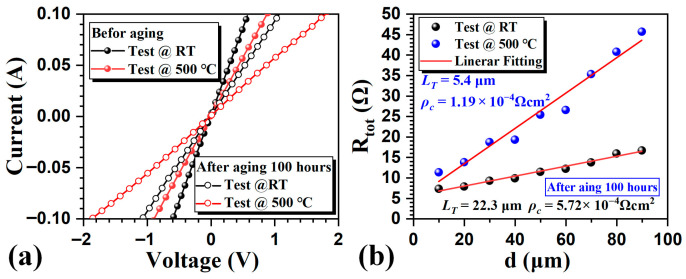
(**a**) *I–V* characteristics (at 20 μm gap) of the W/C stacked Ohmic contacts before and after 100 h aging at RT and 500 °C. (**b**) The total resistance of the W/C stacked Ohmic contacts after 100 h aging as a function of the gap spacing at RT and 500 °C.

**Figure 5:**
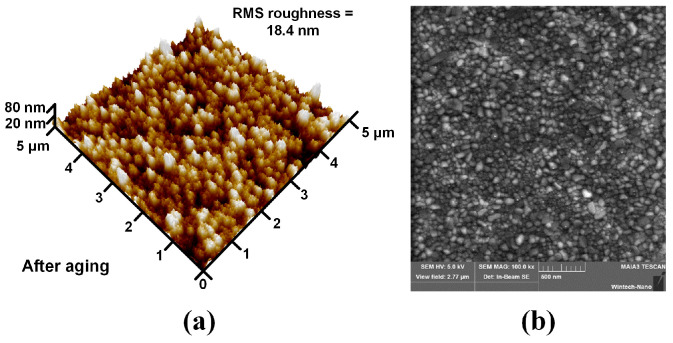
(**a**) Representative AFM surface 3D image of the samples after 100 h aging in the air. (**b**) Top view SEM image of the samples after 100 h aging in the air.

Finally, to evaluate the thermal stability, the samples were aged by heating to 500 °C in a tube furnace with ambient air introduced. Figure 4a shows the *I–V* characteristics before and after 100 h aging at RT and 500 °C. After 100 h of aging, the *I–V* curves maintain ohmic behaviors at both RT and 500 °C. The *ρ*_c_ after 100 h aging was extracted from Figure 4b. There is no slight degeneration in *ρ*_c_ after aging (5.72 × 10^−4^ Ω·cm^2^ at RT and 1.19 × 10^−4^ Ω·cm^2^ at 500 °C). The results show that the fabricated Ohmic contacts are thermally stable. The AFM surface 3D image and top view SEM image of the samples after aging are shown in Figure 5a,b. The root mean square roughness is 18.4 nm, and the crystallization is still uniform after aging. This indicates that such harsh aging conditions do not strongly affect the surface morphology of the contact metal, implying that no interfacial reactions or degradation occurred during the thermal stress process in air, proving that the ohmic contact has good structural thermal stability.

Table 1 summarizes representative relevant excellent work [11,12,13,14,23], including their doping concentrations, aging conditions, and variations in specific contact resistivity. Most previously published works rely on relatively high doping levels by ion implantation process (typically 1 × 10^17^–1 × 10^19^ cm^−3^, denoted by * in the table) and conduct thermal-aging tests predominantly in inert N_2_ or vacuum environments. Under these conditions, Ni contacts exhibit failure after aging at 400 °C. Even multilayer systems, including TiW/Ni, Au/Ni, and Pt/Ti/TiW, show marked increases in *ρ*_c_ after prolonged high-temperature exposure, indicating interfacial reactions or degradation during thermal stress.

In contrast, the W/C stacked Ohmic contact presented in this work is formed directly on a very low doping concentration epitaxial layer (2.5 × 10^15^ cm^−3^) without ion implantation, which is markedly lower than that in previous studies. Despite the more challenging doping condition, the W/C system maintains a moderate increase in *ρ*_c_ (from 2.5 × 10^−4^ Ω·cm^2^ to 5.7 × 10^−4^ Ω·cm^2^) after aging at 500 °C for 100 h in ambient air, which is a much harsher and more application-relevant environment compared with the inert/vacuum atmospheres commonly used. This result suggests the potential robustness of the W/C interface even without protective inert conditions.

## 4. Conclusions

In this paper, we propose a novel method for the fabrication of n-type 4H-SiC Ohmic contact on the low-doped epitaxial layer directly and without extra ion implantation. The novel method uses W/C multi-nanolayers stacked and 1200 °C RTP. The fabricated Ohmic contact shows good specific contact resistance and can work up to 500 °C. The 100 h of long-term thermal stability aging imply the fabricated Ohmic contact can handle the working challenges of extreme heat. Most notably, the novel method without extra ion implantation means low thermal and low manufacturing budgets. This method has great commercial potential for SiC semiconductor devices and integrated circuits.

## Figures and Tables

**Table 1:** Results of N-type SiC Ohmic contact.

Metal	Doping Concentration (×10^15^ cm^−3^)	Aging Conditions	*ρ*_c_ (×10^−5^ Ω·cm^2^) Before/After	Ref.
Ni	5000 *	400 °C/20 h/N_2_	6.0/failed	[12]
TiW/Ni	5000 *	400 °C/100 h/N_2_	4.2/76	[12]
Au/Ni	10,000 *	500 °C/100 h/N_2_	50/500	[11]
Pt/Ti/TiW	11,000 *	500 °C/520 h/N_2_	1.5/50	[13]
W/Ni/Al	100 *	600 °C/300 h/V	N/A	[23]
W/WC/TaC	7800 *	1000 °C/600 h/V	3.0/3.8	[14]
W/C stacked	2.5	500 °C/100 h/Air	25/57	our

* With ion implantation, V is the vacuum.

## Data Availability

The data presented in this study is available on request from the corresponding author. The data is not publicly available due to privacy restrictions.
